# A taxonomic revision of three Chinese spurless species of genus *Epimedium* L. (Berberidaceae)

**DOI:** 10.3897/phytokeys.78.11640

**Published:** 2017-03-23

**Authors:** Shaoxiong Liu, Linjian Liu, Xiaofang Huang, Yuye Zhu, Yanqin Xu

**Affiliations:** 1 College of Pharmacy, Jiangxi University of Traditional Chinese Medicine, Nanchang 330004 P. R. China

**Keywords:** *Epimedium*, spurless, taxonomy, revision

## Abstract

Due to some common or similar features (e.g., small leaf, spurless, yellow flower), three Chinese species of the genus *Epimedium* (Berberidaceae), *E. ecalcaratum*, *E. platypetalum*, and *E. campanulatum*, are controversial based on morphological characteristics. In the present study, the descriptions of morphological characteristics for the three species were revised based on extensive studies and observations both in field and in herbaria. In general, *E. ecalcaratum* has long creeping rhizomes 1–3 mm in diameter, two alternate or opposite trifoliolate leaves, 7–14 flowers, and petals obovate and apex subacute. *Epimedium platypetalum* has short or long-creeping rhizomes 1–3 mm in diameter, one trifoliolate leaf, 2–6 flowers, and petals oblong and apex rounded. *Epimedium campanulatum* has compact rhizomes 4–6 mm in diameter, two alternate or opposite trifoliolate leaves, 15–43 flowers, and petals obovate and apex rounded. Through comparison, we found that despite the close affinity of these three species, they can be distinguished by rhizome differences, stem-leaves, the morphology of flower (e.g., petals), and the number of per inflorenscence.

## Introduction

As a traditional Chinese herbal medicine, *Epimedium* has been widely used for “strengthening the kidney” and “reinforcing bone” for thousands of years (Jiang et al. 2016). Forty-nine species have been reported in China (Ogisu and Rix 2011, Xu et al. 2014, Zhang et al. 2014, Zhang et al. 2015, Zhang et al. 2016, Wei et al. 2017). Stearn (2002) divided the Chinese *Epimedium* into four series: *Campanulatae*, *Davidianae*, *Dolichocerae*, and *Brachycerae*. For a long time, *E. ecalcaratum* G. Y. Zhong, *E. platypetalum* K. I. Meyer, *E. campanulatum* Ogisu and *E. shuichengense* S. Z. He were recognized in the series *Campanulatae* (Stearn 2002). Among them, *E. shuichengense* was once thought to be a very special species with spurless petals, representing a transition stage of floral evolution from series *Campanulatae* (spurless small-flowered species) to series *Davidianae* (long-spurred species) (Stearn 2002). However, according to field investigations in the type locality, Zhang et al. (2015a) confirmed that *E. shuichengense* belongs to series *Davidianae* while *E. reticulatum* C. Y. Wu ex S. Y. Bao truly belongs to series *Campanulatae*. Therefore, four species, namely, *E. reticulatum*, *E. ecalcaratum*, *E. platypetalum*, and *E. campanulatum* were included in the series *Campanulatae* in China.

However, *E. reticulatum* is distinctive and can be easily distinguished from other spurless species. The petals of *E. reticulatum* are flat with a slightly cucullate base, the flower size is obviously smaller (about 7 mm) than other spurless species (about 10 mm), and its leaflets are thickly leathery with conspicuous reticulate veins on both sides (Bao 1987; Zhang et al. 2015a). Therefore, the present study focuses on the remaining three species in series *Companulatae* in China that can easily be confused.

On the other hand, numerous *Epimedium* species have been described without extensive morphological observation. There is also a lack of both field investigations and other studies. *Epimedium ecalcaratum* was described as having compact rhizomes based on very limited samples (Zhong 1991), yet this character was adopted by the *Flora Reipublicae Popularis Sinicae* (Ying 2001). Since *E. platypetalum* was described in 1922, there has been very little research concerning morphological observations for *E. platypetalum*. According to the description based on several individuals grown at Blackthorn Nursery Kilmeston, *E. campanulatum* is morphologically similar to *E. ecalcaratum* and *E. platypetalum* (Ogisu 1996) and it was later treated as a insufficiently known species by Ying et al. (2011). In general, very little is known about the range of variation of characters, variation patterns, and the taxonomic value of these allied species.

Based on extensive studies of the three spurless *Epimedium* species, both in field investigations (during flowering seasons) and in herbaria, the aim of this study was to 1) revise and complete morphological descriptions, and 2) compare the morphological differences among the three similar species.

## Materials and methods

### Field investigation

Field investigations on the germplasm resource and morphological observations have been conducted from 2012 to 2016. Field work was done in Hubei, Shanxi, Chongqing and Sichuan Province, China. A total of 120 individuals (30 individuals per population) from four populations of three spurless species, *E. ecalcaratum* (two populations), *E. platypetalum* and *E. campanulatum*, were collected from Sichuan and Shanxi Provinces (Table 1). All populations were investigated and collected during the flowering, as the floral properties are significant for the taxonomy of *Epimedium* species. To capture variation within populations, 30 individuals per population were observed and sampled. Quantitative measurements on rhizome diameter, height of flowering stem, length of inflorescence, number of flowers, and length and width of the middle leaflet were recorded for each individual. The average data were processed using SPSS 19.0 software. Concurrently, the folloeing discrete morphological characters were observed: the rhizome; pedicel, petiole, underside of leaflet hair characteristics; shape and number of leaflets; number of stem-leaves; inflorescence; leaflets and flowers; shape and color of inner sepals; and shape of petals.

**Table 1.** Location and habitat characters of populations of *E. ecalcaratum*, *E. platypetalum*, and *E. campanulatum*.

Species	Population code	Location (China)	Elevation (m)	Latitude (N)	Longitude (E)	Collect date
*E. ecalcaratum*	SCLD	Longdong, Baoxing, Sichuan	1641	102°44'	30°26'	2015.4.12
	SCBX	Muping, Baoxing, Sichuan	1426	102°50'	30°22'	2015.4.14
*E. platypetalum*	SXLP	Yuanba, Nanzhen, Shanxi	1263	106°36'	32°51'	2016.4.26
*E. campanulatum*	SCLC	Longchi, Dujiangyan, Sichuan	1937	103°35'	31°09'	2016.5.6

### Specimen examination

All 120 individuals of the three species were transplanted at the Jiangxi University of Traditional Chinese Medicine, China. Herbarium specimens were examined from the following herbaria: Chinese Academy of Medical Sciences, Peking Union Medical College Institute of Medicinal Plant Development (IMD); Institute of Botany, Chinese Academy of Sciences (PE); Chongqing Academy of Chinese Materia Medica (SM); Virtual Museum System (HX); Nanjing University (N); and Institute of Botany, Jiangsu Province and Chinese Academy of Sciences (NAS).

## Results

### Geographical distribution

**Figure 1.**
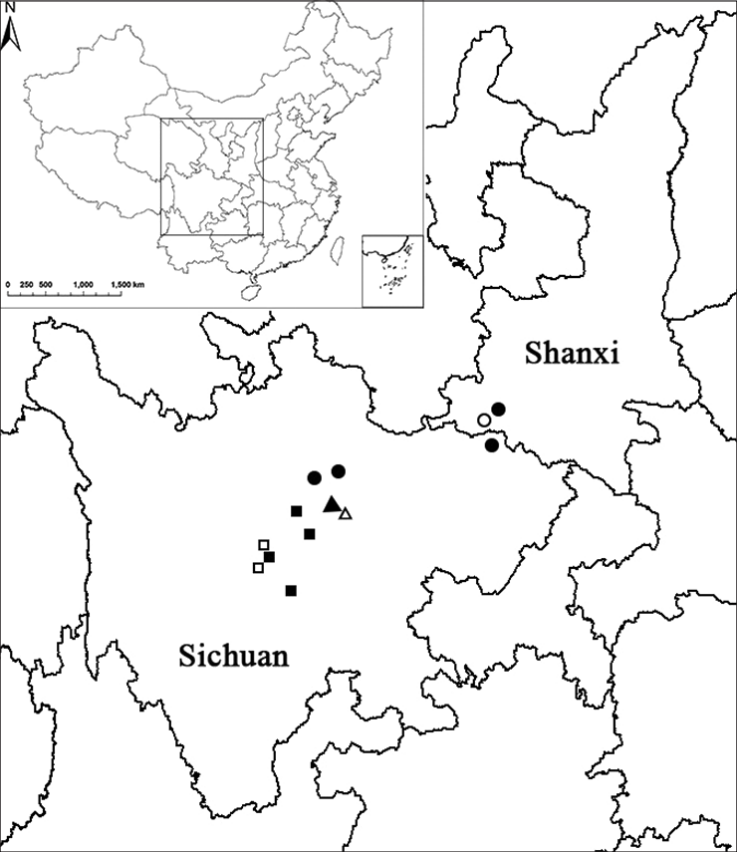
Geographic distribution (black symbols) and sampling populations (white symbols) of *E. ecalcaratum* (square), *E. platypetalum* (circle), and *E. campanulatum* (triangle).

Based on field investigations and herbarium specimens, *E. ecalcaratum*, *E. platypetalum* and *E. campanulatum* were stenochoric species (Fig. 1). *Epimedium platypetalum* has previously been collected in Sichuan; however, it was not observed during our field investigations, likely due to habitat destruction. Therefore, the populations that can be collected were very limited.

### Quantitative characters analysis

Quantitative morphological data from *E. ecalcaratum*, *E. platypetalum*, and *E. campanulatum* are presented in Table 2. Among the three close allies, *E. campanulatum* was easily identified by its long flowering stem and inflorescence, the largest number of flowers, and the stoutest rhizome. *Epimedium ecalcaratum* and *E. platypetalum* were very similar in terms of quantitative traits, but *E. platypetalum* has fewer flowers (2–8 flowers per individual; population mean of four flowers per individual). The main quantitative characters of the two populations of *E. ecalcaratum* were similar, only the length of the flowering stem was found having a slight difference.

**Table 2.** Quantitative traits (mean ± SD) comparative analysis of populations of *E. ecalcaratum*, *E. platypetalum*, and *E. campanulatum*.

Species	*E. ecalcaratum*		*E. platypetalum*	*E. campanulatum*
Population code	SCLD	SCBX	SXLP	SCLC
Height of flowering stem (cm)	25.17±5.43	34.53±6.83	29.73±5.84	42.30±7.70
Length of inflorescence (cm)	12.93±3.40	12.40±2.74	9.46±2.51	17.12±5.84
Number of flowers	9±3	10±2	4±1	25±11
The length of middle leaflet (cm)	3.01±0.58	3.32±0.54	4.35±0.48	5.26±0.71
The width of middle leaflet (cm)	2.30±0.37	2.48±0.44	4.02±0.49	3.37±0.83
The length/ width of middle leaflet	1.31±0.15	1.35±0.12	1.08±0.07	1.62±0.28
Rhizome diameter (mm)	1.71±0.73	1.69±0.33	1.97±0.56	5.12±1.28

### Discrete morphological characters

**Figure 2.**
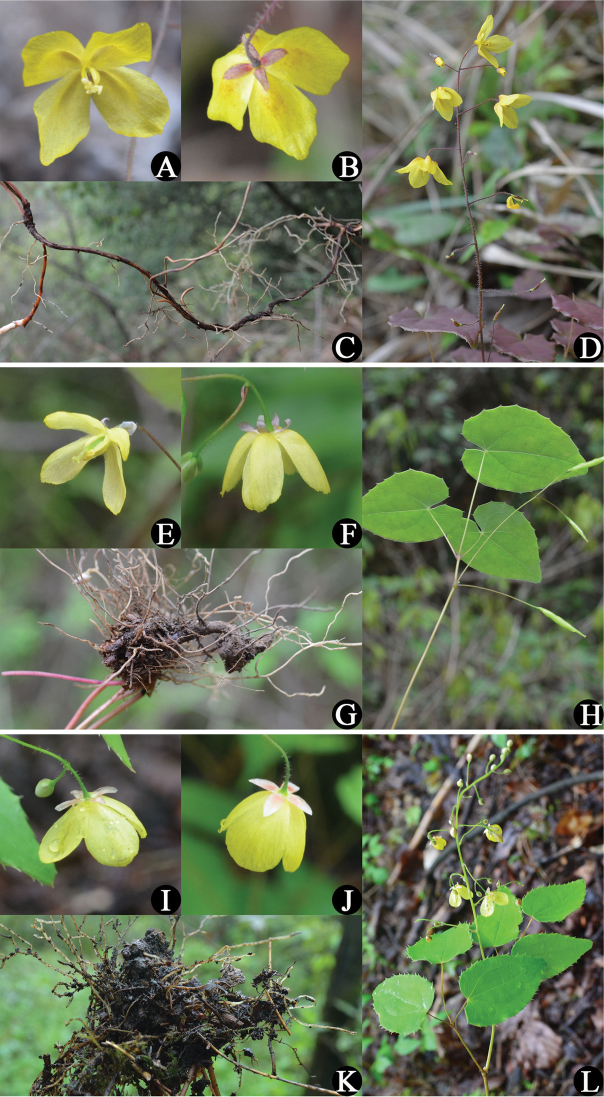
Photos of *E. ecalcaratum*, *E. platypetalum*, and *E. campanulatum*. **A–B** Flower of *E. ecalcaratum* **C** Rhizome of *E. ecalcaratum* **D** Morphology of *E. ecalcaratum* **E–F** Flower of *E. platypetalum* **G** Rhizome of *E. platypetalum* **H** Morphology of *E. platypetalum* **I–J** Flower of *E. campanulatum* **K** Rhizome of *E. campanulatum* **L** Morphology of *E. campanulatum*.

The main discrete morphological characters of *E. ecalcaratum*, *E. platypetalum* and *E. campanulatum* are presented in Table 3. The three species all had glandular hairy inflorescence and pedicels; flat, spurless, yellow, pendulous flowers; and obovate petals. Although having much in common with its close allies, *E. campanulatum* differed by having compound inflorescences and cup-shaped flowers, while *E. ecalcaratum* differed because of the slightly saccate base of the petals, creating a slightly shouldered flower base (Fig. 2). Among the three species, the most diverse characters are the number of leaflets, the number of stem-leaves, and the arrangement of leaves on the stem (Table 3). The rhizome also presented a clear differentiation among the three species.

**Table 3.** The main discrete morphological characters comparative analysis of populations of *E. ecalcaratum*, *E. platypetalum*, and *E. campanulatum*.

Species	*E. ecalcaratum*	*E. platypetalum*	*E. campanulatum*
Shape of petals	Obviate, apex subacute	Oblong, apex rounded	Obviate, apex rounded
Shape of inner sepals	Elliptic	Ovate	Ovate
Colour of inner sepals	Purple-red	Purple-red	Red-tinged
Pedicel indumentum	Glandular hairs	Glandular hairs	Glandular hairs
Inflorescence	Raceme	Raceme	Panicle
Inflorescence indumentum	Glandular hairs	Almost glabrous	Almost glabrous
Shape of leaves	Ovate	Subrounded	Ovate
Blade back indumentum	Pilose	Sparingly pilose	Pilose, vein evident
Petiolule indumentum	Pilose	Glabrous	Almost glabrous
Stem-leaves	Two alternate or opposite trifoliolate leaves	One trifoliolate leaves, sometimes two opposite trifoliolate leaves	Two alternate or opposite trifoliolate/5-foliolate leaves, sometimes three alternate trifoliolate leaves
Rhizome	Long creeping	Thin, short or long-creeping	Compact

### Taxonomic treatment

#### Epimedium ecalcaratum

Taxon classificationPlantaeORDOFAMILIA

G. Y. Zhong

Figs 2A–D , 3A–G

Epimedium ecalcaratum G. Y. Zhong, Acta. Phytotax. Sin., 29: 89. 1991. Type: China. Sichuan: Baoxing, alt. 1100 m, 30 May 1987, *G. Y. Zhong 87-02* (Holotype, SM).

##### Description.

Flowering stem 20–40 cm long. Rhizome long creeping, 1–3 mm in diameter. Leaves basal and cauline, trifoliolate, 5-foliolate, sometimes 7-foliolate; leaflets ovate, 2.5–4 × 2–3 cm, apex acuminate, base deeply cordate with regularly rounded lobes, terminal leaflet with equal rounded lobes, lateral leaflets oblique with outer lobe large and rounded, inner lobe smaller and rounded, margin spinose-serrate, abaxially long-pilose. Flowering stem usually with two alternate or opposite trifoliolate leaves, or sometimes three alternate trifoliolate leaves, occasionally two opposite 5-foliolate leaves, rarely three or four whorled leaves with unifoliolate, trifoliolate and/or 5-foliolate. Inflorescence raceme, 7–14-flowered, 10–16 cm long, glandular hairs. Flowers ca. 10 mm in diam., yellow, pedicels 1–2 cm long, glandular hairy. Outer sepals 4, pale purple, broadly ovate, ca. 4 × 1.5 mm. Inner sepals 4, purple-red, elliptic, ca. 5 × 1.5 mm. Petals 4, yellow, or sometimes purple-red spotted at base, spurless, ca. 6–8 × 4–5 mm, obovate, apex subacute. Stamens ca. 4.5 mm; anthers yellow, ca. 1.5 mm.

##### Distribution and habitat.

*Epimedium ecalcaratum* occurs in Baoxing, Luding, Shimian and Pengxian, Sichuan, often on mountain slopes and grassland with elevations ranging from 1100 m to 2100 m.

##### Phenology.

*Epimedium ecalcaratum* flowers from April to May, and fruits from May to August.

##### IUCN Red List category.

*Epimedium ecalcaratum* was designated as endangered (EN) according to the International Union for Conservation of Nature (IUCN) Red List criteria (IUCN 2015), because of exploitation for medicinal use.

##### Specimens examined.

**China. Sichuan**: Baoxing, *B.L. Guo 0539* (IMD), *D.J. Yu 1892* (PE), *G.Y. Zhong Z-8901* (SM), *Y.Q. Xu & S.X. Liu 2015019* (JXCM), *2016022* (JXCM). Luding, *B.L. Guo 0611* (IMD). Shimian, *Shimian Econ. Pl. Exped. 78-0222* (SM). Pengxian, *Z.B. Feng, D.H. Zhu & X.J. Li, 20070428* (HX).

#### Epimedium platypetalum

Taxon classificationPlantaeORDOFAMILIA

K. I. Meyer

Figs 2E–H , 3H–L

Epimedium platypetalum K. I. Meyer, Repert. Spec. Nov. Regni Veg. Beih., 12: 380. 1922. Type: China. Sichuan: Wenchuan, alt. 1600 m, *Limpricht 1386* (Syntypes, WRSLE, WU).

##### Description.

Flowering stem 25–35 cm long. Rhizome thin, short or long-creeping, 1–3 mm in diameter. Leaves basal and cauline, trifoliolate; leaflets subrounded, ca. 4.5 × 4 cm, apex rounded, base deeply cordate with regularly rounded lobes, terminal leaflet with equal rounded lobes, lateral leaflets oblique with outer lobe large and rounded, inner lobe smaller and rounded, margin spinose-serrate, abaxially pilose. Flowering stem with 1 trifoliolate (rarely 5-foliolate) leaves, sometimes 2 opposite trifoliolate leaves, occasionally 2 opposite unifoliolate leaves. Inflorescence raceme, 2–6-flowered, 7–12 cm long, almost glabrous. Flowers ca. 10 mm in diameter, yellow, pedicels 0.5–1 cm long, glandular hairs. Outer sepals 4, green, triangular-lanceolate, ca. 2 ×1 mm. Inner sepals 4, purple-red, ovate, ca. 4 × 1.5 mm. Petals 4, yellow, spurless, ca. 6–8 × 4–5 mm, oblong, apex rounded. Stamens ca. 3 mm; anthers yellow, ca. 2 mm.

**Figure 3.**
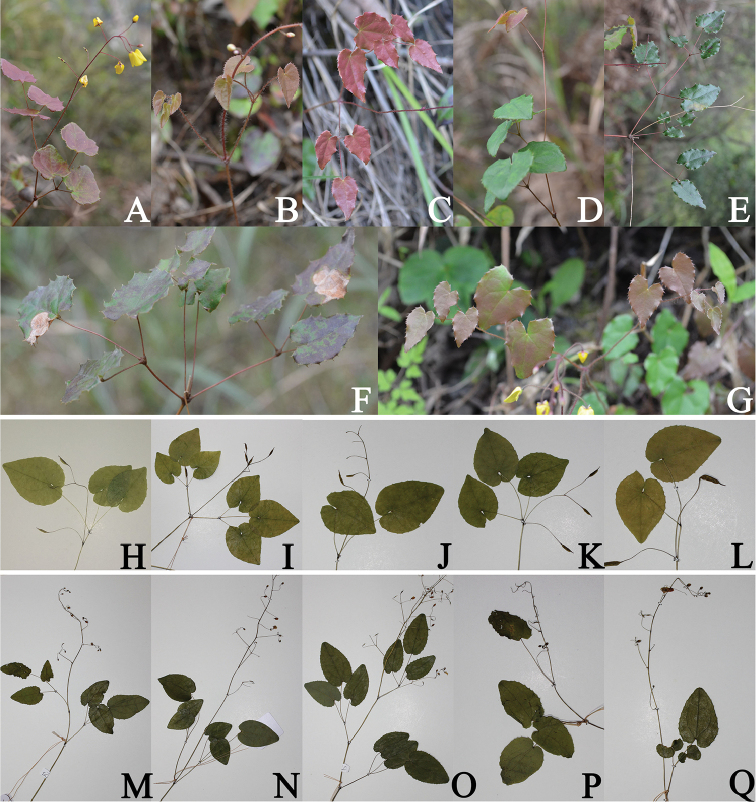
Population variation of leaves morphology in *E. ecalcaratum*, *E. platypetalum*, and *E. campanulatum*. **A–G** *E. ecalcaratum* **H–L** *E. platypetalum* **M–Q** *E. campanulatum*.

##### Distribution and habitat.

*Epimedium platypetalum* occurs in Sichuan and Shanxi, often on mountain slopes with elevations ranging from 1300 m to 2800 m.

##### Phenology.

*Epimedium platypetalum* flowers from April to May, and fruits from May to June.

##### IUCN Red List category.

*Epimedium platypetalum* should be designated as Vulnerable (VU) according to the International Union for Conservation of Nature (IUCN) Red List criteria (IUCN 2015), because of habitat destruction.

##### Specimens examined.

**China. Sichuan**: Maoxian, *S. Y. Chen 5210* (NAS). Nanjiang, *B. L. Guo A56* (IMD). **Shanxi**: Nanzheng, *J.X. Lv, Y.P. Lv & X.F. Zhuo E84009* (PE), *E84010* (PE), *E84052* (PE), *S.X. Liu, J.X. Zhu & L.J. Liu 2016020* (JXCM).

#### Epimedium campanulatum

Taxon classificationPlantaeORDOFAMILIA

Ogisu

Figs 2I–L , 3M–Q

Epimedium campanulatum Ogisu, Kew Bull., 51: 401. 1996. Type: China. Sichuan: Dujiangyan, alt. 2000 m, 15 May 1994, *Ogisu 94305* (Holotype, K).

##### Description.

Flowering stem 35–50 cm long. Rhizome compact, 4–6 mm in diameter. Leaves basal and cauline, trifoliolate; leaflets ovate, 4.5–6 × 2.5–4 cm, vein evident, apex rounded, base deeply cordate with regularly rounded lobes, terminal leaflet with equal rounded lobes, lateral leaflets oblique with outer lobe large and rounded, inner lobe smaller and rounded, margin spinose-serrate, abaxially pilose. Flowering stem usually with 2 alternate (mostly 2 trifoliolate and occasional 1 trifoliolate and 1 unifoliolate) or opposite leaves, sometimes 3 alternate leaves, and 1 trifoliolate leaves also occasionally observed. Inflorescence panicle, 15–43-flowered, 11–23 cm long, almost glabrous. Flowers ca. 10 mm in diam., yellow, pedicels 1.2–1.8 cm long, glandular hairs. Outer sepals 4, green, broadly ovate, ca. 2 ×2.5 mm. Inner sepals 4, red-tinged, ovate, ca. 2.5–3 × 1–1.5 mm. Petals 4, yellow, spurless, ca. 6–8 × 5–7 mm, obovate, apex rounded. Stamens ca. 2.5 mm; anthers yellow, ca. 1 mm.

##### Distribution and habitat.

*Epimedium campanulatum* occurs in Dujiangyan, Sichuan, often on mountain slopes; 2000 m.

##### Phenology.

*Epimedium campanulatum* flowers from May to June, and fruits from June to July.

##### IUCN Red List category.

Only known from Dujiangyan, Sichuan, *Epimedium campanulatum* was designated as endangered (EN) according to the International Union for Conservation of Nature (IUCN) Red List criteria (IUCN 2015), because of environment deterioration.

##### Specimens examined.

**China. Sichuan**: Dujiangyan, *Q. Wang, K. Yao, 9015* (PE), *Z.B. Feng 960035* (HX), *C. Zhang, Z.B. Feng 960055* (HX), *C. Zhang, G.J. Wu 990210* (HX), *S.X. Liu & L.J. Liu 2016028* (JXCM), *T.T. Yu 801* (N).

## Discussion

### Key to species of ser. Campanulatae

**Table:** 

1	Flower ca. 7 mm; petals are flat with slightly cucullate base; leaflets are thickly leathery	***E. reticulatum* C. Y. Wu ex S. Y. Bao**
–	Flower ca. 10 mm; petals are flat; leaflets are membranaceous.
2	Rhizome compact, diameter 4–6 mm; inflorescence panicle	***E. campanulatum* Ogisu**
–	Rhizome long creeping, diameter 1–3 mm; inflorescence raceme.
3	Inflorescence usually 7–14-Flowered, two alternate or opposite trifoliolate leaves	***E. ecalcaratum* G. Y. Zhong**
–	Inflorescence usually 2–6-Flowered, one trifoliolate leaf, sometimes two opposite trifoliolate leaves	***E. platypetalum* K. Meyer**

The protologue (Zhong 1991) and the subsequent description in *Flora of China* (Ying 2001) both described the compact rhizome of *E. ecalcaratum*. We re-examined the holotype and conducted fieldwork in its type locality. However, individuals of *E. ecalcaratum* in the field all had long creeping rhizomes, slender nodes with numerous fibrous roots, 1–3 mm in diameter, and internodes sometimes to 30 cm. In the genus *Epimedium*, this situation may not be rare. The form of the rhizome, specifically the degree of elongation and thickness, and also the average size of the terminal winter-bud, is constant for each species, and sometimes offers contrasts of taxonomic value (Stearn 2002). But Stearn (1997) pointed out that the different rhizome forms among some *Epimedium* species can sometimes be very evident, sometimes more subtle. For example, the major difference between *E. leptorrhizum* Stearn and *E. brachyrrhizum* Stearn was that the former had a very slender elongated rhizome while the latter bore a more compact clump-forming rhizome. However, examination of a series of *E. leptorrhizum* specimens showed that its rhizome was often slender and long-creeping but occasionally thicker and compact (Zhang et al. 2015b). In addition, the protologue of *E. lishihchenii* Stearn differs from *E. franchetii* Stearn was in having a long-creeping rhizome (Stearn 1997). However, our field observation based on a population found that *E. lishihchenii* 20% of individuals had compact rhizomes (Liu et al. 2016).

Due to the slender elongated rhizome and small broadly ovate or almost orbicular leaflets, Guo et al. (1993) described a variety, E. platypetalum var. tenuis B. L. Guo et P. G. Hsiao. Stearn (1995) assessed E. platypetalum var. tenuis and found it differed from *E. platypetalum* bacause of its long-spurred flowers. Then, he treated E. platypetalum var. tenuis as a synonym of *E. pauciflorum* K. C. Yen, a new species that was published after the description of E. platypetalum var. tenuis (Yen 1994; Stearn 1995). In a more recent study, Ying et al. (2011) still treated E. platypetalum var. tenuis as a synonym of *E. platypetalum*. We re-examined the specimens of E. platypetalum var. tenuis, and found the taxon had obviously long-spurred petals (1.7 cm) and long inner sepals (1.4 cm) that were completely different with *E. platypetalum*. In addition, E. platypetalum var. tenuis has sympatric distribution with *E. pauciflorum*. Therefore, we agree with Stearn (1995) that E. platypetalum var. tenuis should be revised as a synonym of *E. pauciflorum*.

The number of stem-leaves was believed to be stable within a species and important for taxonomy, and three informal groups have been divided by the normal number of stem-leaves (Stearn 2002). Stem-leaves, however, are not so unvarying as initially supposed. Stearn (2002), Zhang et al. (2015), and our field observation have recognized and recorded some variation on the number of leaves. For example, usually two opposite or occasionally three whorled leaves were observed in *E. sagittatum* (Sieb. et Zucc.) Maxim., *E. acuminatum* Franch., *E. myrianthum* Stearn and *E. franchetii*, and one leaf or two leaves in *E. epsteinii* Stearn, *E. flavum* Stearn, *E. leptorrhizum* and *E. pauciflorum*.

It is significant that the comparatively unstable species occur in western China, where the genus is best represented and where its evolution may still be proceeding (Stearn 2002). Population is the basic unit of evolution (Chen 2016) and the most important unit to study the formation of species (Chen and Wang 1986; Nooteboom 1992; Hong 2016). Thus, morphological differences recorded among individuals in a population should not be ignored and may be more obvious than populations in sometimes (Yang 1991; Jonas et al. 1999). Previous studies on the taxonomy of the genus *Epimedium* were almost always based on limited samples, or several individuals cultivated abroad (Ying 2001). Our investigations based on populations in their native habitat found that the number of leaves and the habit of the flowering stem presented abundant variation (Fig. 3).

The protologue for *E. ecalcaratum* described that its stem-leaves are usually opposite with two trifoliolate leaves, occasionally alternate with two trifoliolate leaves or three trifoliolate leaves (Zhong 1991). Our investigation showed that it usually had two alternate or opposite trifoliolate leaves, sometimes three alternate trifoliolate leaves, occasionally two opposite 5-foliolate leaves, or rarely three or four whorled unifoliolate, trifoliolate and/or 5-foliolate leaves (Fig. 3A–G). In the protologue of *E. campanulatum* (Ogisu 1996), the species was described with one leaf or two usually alternate, rarely opposite leaves, but our investigation showed that it usually had two alternate (mostly two trifoliolate, and occasionally one trifoliolate and one simple) or opposite leaves, sometimes three alternate leaves, and one trifoliolate leaf was also occasionally observed (Fig. 3M–Q). Our study clearly shows that the number and insertion of the leaves and the number of leaflets varies in this species. Upon extensive specimen examination, Zhang et al. (2011, 2015b) observed that the leaves of *E. simplicifolium* T. S. Ying were mainly unifoliolate, occasionally trifoliolate, and the leaves of *E. acuminatum* Franch. may be unifoliolate. Subsequently, *E. simplicifolium* was synonymized with *E. acuminatum* (Zhang et al. 2011). Hence, as a taxonomist, it is important to study as many collections as possible (Xu 1998).

Although having much in common with *E. platypetalum* and *E. campanulatum*, *E. ecalcaratum* differs in having slightly a saccate petal base, creating a slightly shouldered base to the flower (Stearn 2002), which is in agreement with our field observations (fig. 2A, B, D). According to Stearn’s research, the character could be regarded as moving towards development of a nectar-producing spur, and he published a photo to show the petals are typically without a spur, but may have varying degrees of small spurs (Stearn 2002: 53, fig. 18). This may indicate that *E. ecalcaratum* represented a transitional stage in floral evolution from series *Campanulatae* (spurless) to series *Davidiance* (spur with basal lamina). Spur variations have not been observed in the present study, and are not supported by specimens or other literature.

## Conclusions

Despite similarity in leaf size and flat, suprless, yellow flowers, *E. ecalcaratum*, *E. platypetalum* and *E. campanulatum* could be distinguished by the following characters: rhizome form, number of stem-leaves, leaflets, flowers, inflorescence, and petals and inner sepal shape (Table 4 and Fig. 2).

**Table 4.** Comparison of key characteristics of *E. ecalcaratum*, *E. platypetalum*, and *E. campanulatum*.

Species	*E. ecalcaratum*	*E. platypetalum*	*E. campanulatum*
Shape of petals	Obovate, apex subacute	Oblong, apex rounded	Obovate, apex rounded
Inner sepals	Purple-red, elliptic	Purple-red, ovate	Red-tinged, ovate
Number of flowers	7–14	2–6	15–43
Inflorescence	Raceme, 10–16 cm	Raceme, 7–12 cm	Panicle, 11–23 cm
Leaves	Ovate, 2.5–4 × 2–3 cm	Subrounded, ca. 4.5 × 4 cm	Ovate, 4.5–6 × 2.5–4 cm
Stem-leaves	Two alternate or opposite trifoliolate leaves	One trifoliolate leaves, sometimes two opposite trifoliolate leaves	Two alternate or opposite trifoliolate/5-foliolate leaves, sometimes three alternate trifoliolate leaves
Rhizome	Long creeping, 1–3 mm	Thin, short or long-creeping, 1–3 mm	Compact, 4–6 mm

## Supplementary Material

XML Treatment for Epimedium ecalcaratum

XML Treatment for Epimedium platypetalum

XML Treatment for Epimedium campanulatum
